# Investigation of Compression and Buckling Properties of a Novel Surface-Based Lattice Structure Manufactured Using Multi Jet Fusion Technology

**DOI:** 10.3390/ma14102599

**Published:** 2021-05-17

**Authors:** Aamer Nazir, Mubasher Ali, Jeng-Ywan Jeng

**Affiliations:** 1 Department of Mechanical Engineering, National Taiwan University of Science and Technology, Keelung Road, Taipei 106, Taiwan; aamernazir.an@mail.ntust.edu.tw; 2 High Speed 3D Printing Research Center, National Taiwan University of Science and Technology, Keelung Road, Taipei 106, Taiwan; Mubashersuit.edu.pk@gmail.com; 3 President Office, Lunghwa University of Science and Technology, No.300, Sec.1, Wanshou Rd. Guishan District, Taoyuan City 333326, Taiwan

**Keywords:** Additive Manufacturing (AM), design for AM, lattice structures, energy absorption, structural behavior, critical buckling load, specific strength

## Abstract

Lattice structures possess many superior properties over solid materials and conventional structures. Application-oriented lattice structure designs have become a choice in many industries, such as aerospace, automotive applications, construction, biomedical applications, and footwear. However, numerical and empirical analyses are required to predict mechanical behavior under different boundary conditions. In this article, a novel surface-based structure named O-surface structure is designed and inspired by existing Triply Periodic Minimal Surface morphologies in a particular sea urchin structure. For comparison, both structures were designed with two different height configurations and investigated for mechanical performance in terms of compression, local buckling, global buckling, and post-buckling behavior. Both simulation and experimental methods were carried out to reveal these aforementioned properties of samples fabricated by multi jet fusion technology. The sea urchin structure exhibited better mechanical strength than its counterpart, with the same relative density almost two-folds higher in the compressive response. However, the O-surface structure recorded more excellent energy absorption and flexible behavior under compression. Additionally, the compression behavior of the O-surface structure was progressive from top to bottom. In contrast, the sea urchin structure was collapsed randomly due to originated cracks from unit cells’ centers with local buckling effects. Moreover, the buckling direction of structures in long columns was also affected by keeping the relative density constant. Finally, based on specific strength, the O-surface structure exhibited 16-folds higher specific strength than the sea urchin structure.

## 1. Introduction

Improvement in mechanical properties such as energy absorption, compression behavior, or stiffness of additively manufactured (AM) lattice structures has been carried out in various ways, including the structure and material optimization and selection of fabrication technologies. However, investigation and design of a lattice structure related to the physical system emerged rapidly during the past decade [1,2,3]. This essential realization through new lattice structure topology design is a fundamental process to understand the materials and structural behavior such as the design of a single lattice structure with variable parameters, the design of a new lattice structure, or topology optimization [4,5,6]. Various aspects of lattice structure need to be considered to improve these mechanical properties, such as relative density, wall thickness, cell size, or strut dimeter. However, its end-use depends upon the specific preferences of each industry. Almost all the novel lattice structures are inspired by nature to optimize their structures’ morphology [7,8].

The lattice structure is an architecture formed by an array of unit cells’ spatial arrangements with edges and faces. The plates and struts form the faces and edges of the cells. Lattice structures exist in two main topologies: stochastic and periodic. There is no systematic arrangement of the unit cell in the stochastic lattice structure while, in a regular lattice structure, the cells are arranged along a separate axis [9]. These cellular structures exist in different types, such as honeycombs, lattices, and foams [10,11]. They can be beam-based or wall-based structures, such as Octet, Kelvin and Gyroid, Diamond, Split-P, respectively. Its application can be divided into end-industry goals, including structural, functional, or ergonomic parts [12]. More specifically, biomedical [13], thermal [14], and materials-improvement properties [15].

Lattice structures are an excellent choice for applications where stiffness is required to be as high as possible for a given mass. In this regard, new structures were designed and investigated to enhance stiffness properties [16,17,18]. Kumar et al. [19] developed a new structure inspired by a sea urchin and studied its manufacturability of close and open cells without support. The author claimed that the smaller size of cell size exhibited better stiffness, and the design is fail-safe despite the same relative density. Maskery et al. [20] also numerically investigated five different kinds of lattice structures, functionally graded by tailored volume friction. They found that the I-WP lattice structure recorded the highest stiffness in one loading direction. However, the diamond structure also showed a greater isotropic behavior. A lattice structure with an interior column contributes to more excellent mechanical properties instead of its outer shell [21]. Column-based lattice structures can further be designed with hollow or solid columns. In a hollow column, the column probably has a higher diameter for wall-based lattices, while solid columns are usually designed for beam-based lattice structures [22]. However, mainly cellular structures are influenced by the following three properties: relative density, the structure’s geometry, and the unit cell size/number of the unit cells with material properties [9,23]. Saghaian et al. [24] systematically investigated three different additively manufactured NiTi triple periodic minimal surface structures in terms of material properties and morphology with a 69% constant porosity level. As a result, the mechanical properties of porous samples were highly dependent on the structure geometry. Additionally, it was mentioned that gyroid and diamond structures are suitable for high strain, while the Schwartz structure was recommended for low-stress levels. Primo et al. [25] optimized and compared the C-Clip geometry by an innovative lattice topology approach by minimizing its overall mass. The author aimed to improve the mechanical properties by comparing and reducing the structure’s mass. These novel designed and compared lattice structures proved to have the best mechanical, electrical, heat-mass-transfer, and biomedical properties [26,27,28,29]. Latture et al. [30] compared additively manufactured octet truss structures with fillets and without fillets at nodes for the compressive response. Meanwhile, the relative densities did not remain the same, and the structures with fillets had a 12% higher relative density than their counterpart. Moreover, a high performance-to-weight ratio is also a popular feature of lattice structures. Arshad et al. [22] improved the failure mechanism, absorption energy, and stiffness by altering beam-based lattice structures and changing the cell topologies with constant relative density. To keep the relative density constant, it is necessary to consider the sufficient material present in the load areas [31]. Apart from high stiffness, compressive and energy-absorption properties are also highly desired by lattice structures [30,31].

Buckling is one of the significant causes of machine elements’ failure, and many structures require its stability. Therefore, the possibility of buckling should always be considered in the designing phase, and much care needs to be given to critical buckling. A wide range of studies were conducted to buckle beams, planes, and shells [32,33,34]. However, to investigate critical buckling of lattice structures, Nazir et al. [35] studied the variable-density lattice structure columns for critical and post-buckling behavior. They revealed that critical buckling depends on several parameters, such as the thickness of the beam, orientation, second moment of inertia, and mass distribution in the structures. Additionally, crack propagation can happen if these parameters are considered inappropriate [36]. In outcomes, they claimed that designing a variable-density cellular column can enhance its critical buckling. These parameters and structure morphology also play a vital role in affecting the buckling of lattice structures. In order to determine the critical buckling load, optimal parameters need to be identified for respective applications. However, Qianqian et al. [37] also proved by a numerical and analytical study that the column’s buckling mode could significantly depend upon its height.

Most previous work on lattice structures was conducted to increase their mechanical properties, such as compressive properties [38,39]. However, countable researchers have investigated surface-based lattice structures. Similarly, the lattice structure column was studied with limited numbers, whereas holes/pores and elliptical shape-based columns were studied extensively [33,34,35]. Considering these research gaps, a novel lattice design named O-Surface (OS) was introduced and compared with the existing Sea Urchin structure (SU) to investigate and reveal its inherent mechanical properties. However, being a new design, no published data of OS structure exist so far. Furthermore, finite element analysis and experimental work were also carried out to analyze critically the mass and wall thickness effect on mechanical properties, such as compressive strength, fracture behavior, critical buckling load, and energy absorption. Three specimens of each sample were designed by Creo Parametric software and additively manufactured by the HP MJF 580 3D printer (Palo Alto, CA, USA). Compressive testing was conducted according to the ASTM D1621-16 on the MTS Insight 10. Finally, the effect of mass and wall thickness was investigated, and each structure was recommended according to its mechanical properties.

The article proceeds as follows. In Section 1, a general introduction of the work and literature review was summarized with insight into lattice structure investigation and buckling study. The review revealed that although the different lattice structures have been investigated by numerous scientific researchers, very few considered the effect of combined mass and wall thickness in a single study. The review also showed that only a few researchers considered additively manufactured lattice structure columns for buckling, here termed critical buckling. Building on these insights, Section 2 explains the materials and methodology, identifying design strategies of OS and materials properties. In this section, a discussion of experimental work, such as that on compressive properties, is carried out to include the final results. Section 3 assesses and compares the results for outcomes and limitations with recommendations. In the last section, Section 4, conclusions are discussed extensively.

## 2. Material and Methodology

### 2.1. Samples Design

**Figure 1:**
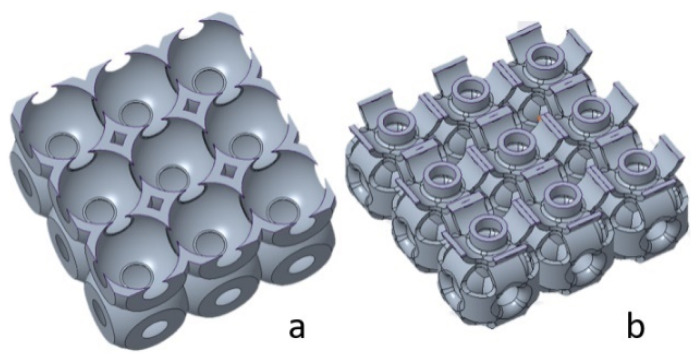
Basic design mechanism of (**a**) SU structure (**b**) O-Surface structure.

In this study, two structures, SU and OS, were designed to investigate their mechanical properties with respect to the mass and wall thickness of the structures. Additionally, comparatively and analyzing the specific strength of these mentioned structures, these two structures were designed based on two strategies: constant mass and constant wall thickness. Not all systems are required to install lightweight to high-strength structures; some are needed with high strength to achieve higher mass. Therefore, in such cases, structure topology needs to be considered. For example, the MeyGen, the world’s largest tidal array, installed a turbine under the water—each turbine stands on a beta swing between 260 to 350 tons—with a mass of 1200 tons. In this system, the turbine’s basement structure was required to be strong enough to withstand turbine mass, and ocean waves with a water-free moving topology. In this case, a beam or wall-based lattice structure with greater mass and strength can be suggested to bear the weight and allow the water waves without vibrating the turbines. For this study, the overall structure idea was inspired by the SU structure in Figure 1a; however, columns are generated based on related reviews [22,35]. The OS structure is a novel lattice structure to investigate its inherent mechanical properties compared to the existing SU lattice structure. The OS structure (see Figure 1b) is designed with the hollow column in the center from six sides, unlike Figure 1a. The newly designed structure is capable of bearing the vertical compression load on the outer shells and generated columns, while the SU can bear this load only on the outer shells. Additionally, OS columns are designed to investigate the critical buckling load of these structures.

**Figure 2:**
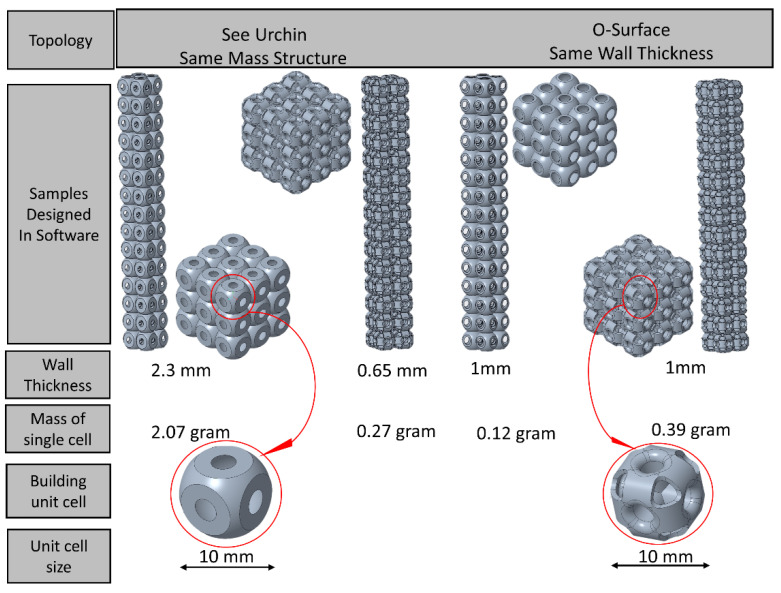
Pictorial representation of structures based on mass and wall thickness.

A 3D modeling software named Creo parametric version 4.0 (1987 Boston, MA, USA) was used to design these structures, and the dimensions and design parameters are listed in Table 1 [40]. The step-wise designing procedure was followed—from a single unit cell of 10 mm to the complete 3 × 3 × 3 and 2 × 2 × 15 structures (Figure 2). The unit size of all the structures and their bounding box of each unit cell were kept constant. In Table 1, the structures in the first section were designed with the same relative density—0.27 g for each structure unit cell—whereas a difference can be seen in the wall thickness. In contrast, for section two, the wall thickness was kept constant (1 mm) with a variation in relative densities. The size of complete structures was too large for tessellation. Therefore, an assembly option was used for it. Three samples for each study—SU, OS, and Column—were designed as shown in Figure 2, which also represents the tessellation of complete structures from a single unit cell. It also represented both structures with the left side—the same mass structures—and the right side—the same wall thickness structures. These structures were designed according to the dimensions given in Table 1 and on both the criterion of constant mass (M) and that of constant wall thickness (T).

### 2.2. Additive Manufacturing and Testing

Polyamide plastic (PA 12) material is widely used in the defense, automotive, and aerospace industries due to its excellent properties. Fabrication of samples with nylon materials revealed the advantages of mechanical properties, such as printing relatively thin, flexible joints [41]. The Scanning electron microscope (SEM) micrographs’ details can be found in the picture with magnification in a previous study [42]. The PA 12 materials’ linear properties mentioned in an earlier study [22] were also used in the present study. Testing was carried out by the MTS universal testing machine (MTS 62 System Corporation, Eden Prairie, MN, USA) with a 10 KN load cell.

The five tensile specimens’ resulting nominal and true stress-strain curves are considered from the authors’ previous study [42]. The elastic and plastic nominal stress-strain data up to the tensile tests’ necking were converted to true stress-strain data using the equations depicted in [43]. Table 2 and Table 3 list the linear and nonlinear data and are assigned to finite element analysis (FEA) to define the PA12 elastic and post-yielding behavior as required by the ANSYS material model.

**Figure 3:**
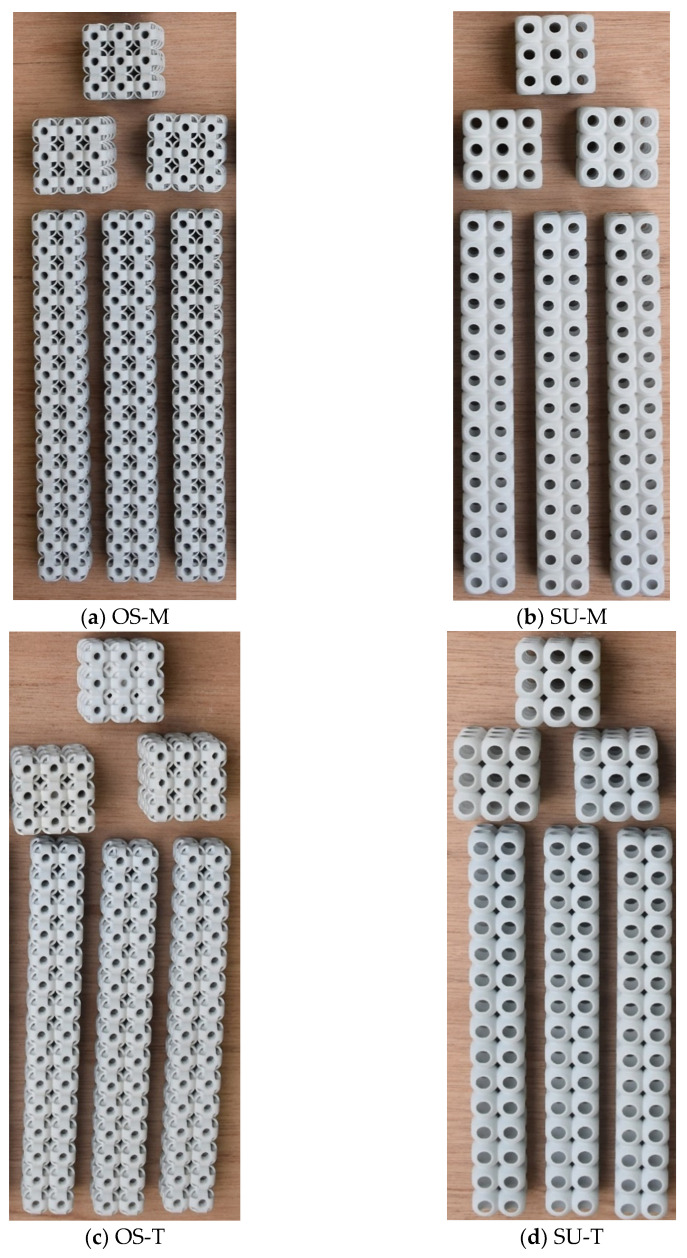
Three specimens of each sample were printed by additive manufacturing (Multi Jet Fusion 3D Printer). (**a**) OS structure with constant mass; (**b**) SU structure with constant mass; (**c**) OS structure with constant wall thickness; (**d**) SU structure with constant wall thickness.

**Figure 4:**
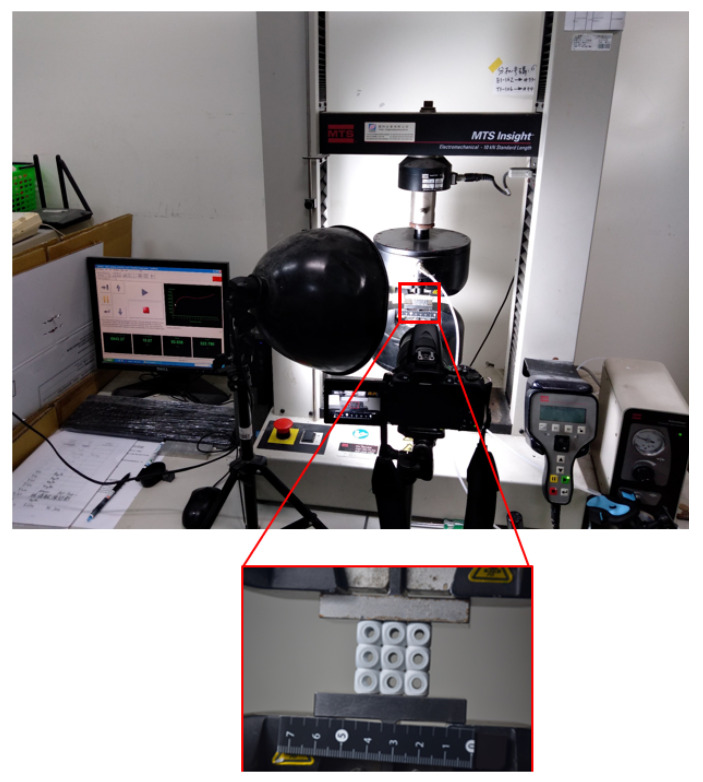
Uniaxial compression testing setup.

**Figure 5:**
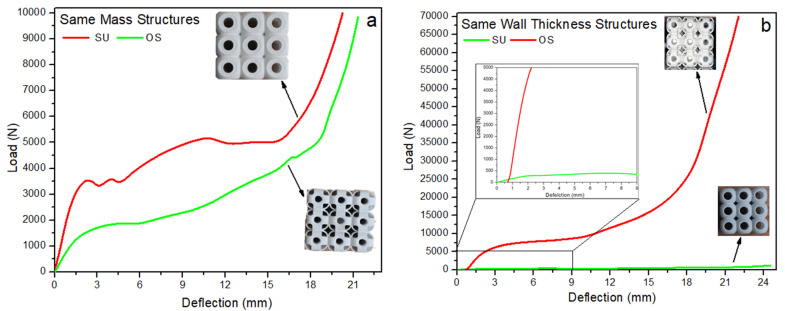
Compressive performance of both 3 × 3 × 3 structures under uniaxial load, (**a**) structure with same relative densities, (**b**) structure with the same wall thickness with an enlarged view of minimum.

In this study, all 24 specimens were fabricated by a multi jet fusion 580 3D printer (HP, Palo Alto, CA, USA) using PA12 materials [44]. Multi jet fusion fabrication and post-processing details can be found here [42]. All samples were printed in the same printing batch, same layer height (80 μm), and same speed (1817 cm^3^/h), and were placed near the center of the build unit in the same orientation to minimize the effects of the printing parameters. Three specimens were fabricated for each sample and can be seen in Figure 3. The mass of all the AM samples was recorded as the same (±5 g) compared to the CAD design. The reason for the increase in mass was powder sticking in the structures. Powder sticking inside the structures is the main reason for it, as explained in the coming sections. All the AM samples were compressed uniaxially according to the ASTM D1621-16 [45] using the universal testing machine, as shown in Figure 4. The MTS machine (MTS Systems Corporation, Eden Prairie, MN, USA) was used to investigate the deflection versus loading, energy absorption, and samples’ failure mechanism. The specimens were tested with a machine loading capacity of 10 kN and a crosshead speed of 10% (3 mm/min) of the total height of samples. However, another higher compression capacity—100 kN—computer-controlled 810 MTS machine (MTS Systems Corporation, Eden Prairie, MN, USA) was used to test the high strength structures [44]. The complete experimental setup is shown in Figure 5.

## 3. Results and Discussion

The results are evaluated for mechanical properties, such as compression, stiffness, and critical buckling load. Additionally, the failure mechanism is also reported for both structures. Stiffness is the slope of stress versus strain value in the elastic region or the force-deflection graph’s peak force before starting the yielding point. However, before the densification begins, the area under the load-deflection is regarded as the actual energy absorbed per unit volume of the lattice structure.

### 3.1. Deformation and Fracture of 3 × 3 × 3 Structures

**Figure 6:**
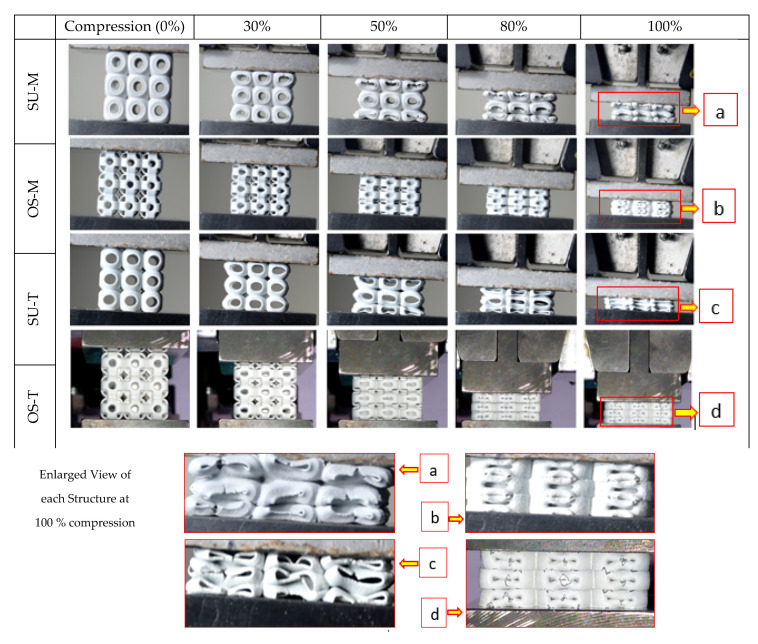
Performance of SU and OS structures with M and T at various stages under progressive compression—from left to the right side—(above), enlarged complete densification stage of each structure shown failure mechanism (below). (**a**) SU-M structure at 100% compression; (**b**) OS-M structure at 100% compression; (**c**) SU-T structure at 100% compression; (**d**) OS-T structure at 100% compression.

This section explains the compressive behavior of the 3 × 3 × 3 SU and OS lattice structures for both the criterion of constant mass (M) and that of constant wall thickness (T). These structures are compressed uniaxially from top to down progressively until the densification stage. The compression behavior for load-deflection is shown with individual figures in Figure 5a,b. Having the same density of both structures, SU-M showed better mechanical strength than OS-M. This significant force is almost two-folds higher in compressive response. Of course, the mass was adjusted by varying the wall’s thickness, as shown in Table 1. However, the OS-M structure failure mechanism was relatively smooth compared to the SU-M structure. The SU-M line of compression showed fluctuation due to the structure’s local buckling—see Figure 6a—at 50% compression. There is no local buckling and crack propagation reported in the OS-M structure under compression. Moreover, OS-M also showed better energy absorption behavior as it bore the force until 19 mm before densification. In contrast, SU-M starts densification before 18 mm.

These structures were also investigated with the same wall thickness; OS-T exhibited higher and significant compressive strength. The enlarged view in Figure 5b shows the considerable difference between the strength to deflection behavior of both structures. This significant difference in the strength may be due to the supported high compressive load on the vertical hollow column in the OS structure, as shown in Figure 2. Additionally, this difference can be justified from Figure 6, where the OS-T structure did not produce any local and global buckling. Moreover, there is no crack propagation seen during its progressive compression.

**Figure 7:**
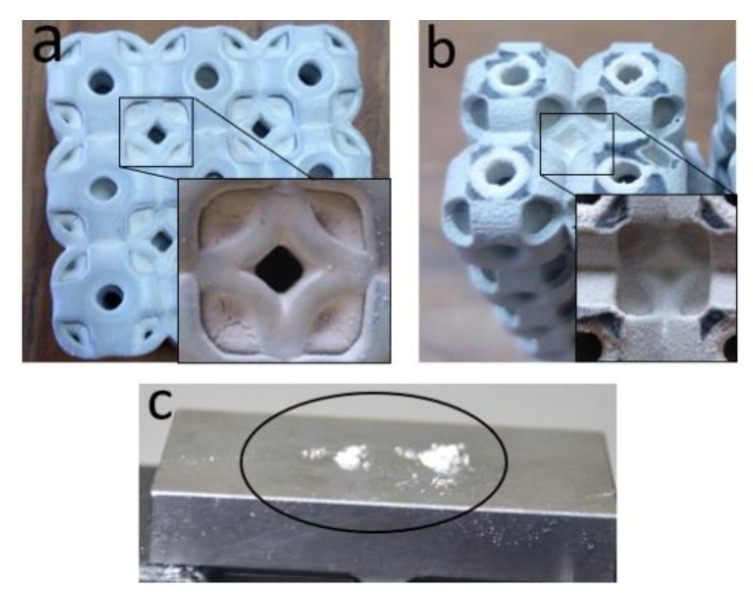
The picture shows the powder presence in the OS-T structures after mechanical testing, (**a**) SU 3 × 3 × 3, (**b**) OS 2 × 2 × 15 structure, (**c**) some quantity of powder removed after compression testing.

In Figure 5b, the OS-T’s compressive strength is more than 20-folds higher than the SU-T with a difference in mass; however, both the structures’ thickness remained the same. Up to ten-folds of the significant improvement in mechanical compressive strength were expected due to the same wall thickness, which increased the relative density by two-folds. However, some of the increase in the stiffness value can be the reason for powder sticking in the OS-T structure. The powder sticking problem can be seen in Figure 7 in which Figure 7a,b showed the stick powder inside the structures after compression, whereas Figure 7c revealed unstuck powder on the compression plate. The overall comparison of both structures with M and T criterion can be observed in Figure 5. These graphs can recommend having the same density of both structures: The SU structure can exhibit higher mechanical strength with rigid compression. At the same time, the OS structure can be preferred for excellent energy absorption. In contrast, to keep the wall thickness constant without considering the structure’s mass, the OS-T is highly recommended due to its significantly higher mechanical strength. The OS structure can also be recommended based on stable physical behavior (see Figure 6b,d).

Failures of the structure are a primary mechanism that significantly influences the mechanical properties, especially under compression. Figure 6 recorded the failure mechanism of the 3 × 3 × 3 SU and OS structures under compression load, such as local buckling of the unit cell and progressive collapsing of the structure. Both the SU samples started compression (30%) by inducing local buckling in the top and bottom unit cell. In comparison, the central unit cell maintained its physical shape until the compression reached 80%. After that, the complete structure started progressively collapsing; however, no significant cracks can be found in the middle of the unit cell as shown in Figure 6a. In contrast, the SU sample with T crashed with cracks due to its low wall thickness.

The OS structure failure mechanism was completely different from the SU structure. The OS structure transfers the load progressively from top to bottom, and all the unit cells are compressed equally consistently. Additionally, this trend towards elastic-brittle failure was compared with elastic-plastic failure due to vertical hollow beams. The steady compression of OS structures can be seen in Figure 6b,d at 30–80%. The OS structure’s vertical columns are the main reason that prevented the OS structure unit cell from cracking. These columns can be seen in Figure 1b, which can support the structure’s vertical downward applied load. These beams imparted great strength to the structure at the cost of energy absorption. There was a brittle failure in the cell’s interior. The vertical beams are ripped as the cells expand laterally under compression and as the vertical beams eliminate the local buckling in the unit cells. In contrast, no columns can be seen in the SU structure to bear the applied load directly, while only the outer shell can withstand the applied load (see Figure 1a). Therefore, all the rows of the OS structure are compressed equally without cracking and crashing. However, it spreads outwards after 95% of compression (see Figure 6b,d at 100%). OS structures behaved in a brittle manner but still showed a progressive cell collapse mechanism. In the enlarged view of Figure 6, both SU structure samples collapsed because cracks originate from the cell’s center due to no support in the middle. In comparison, OS samples collapsed due to cracks initiated from the round corners of the cell instead of the center. This difference in failure mechanisms led to the OS-T structure to be higher in stiffness under compression.

### 3.2. Buckling and Fracture of 2 × 2 × 15 Structure

**Figure 8:**
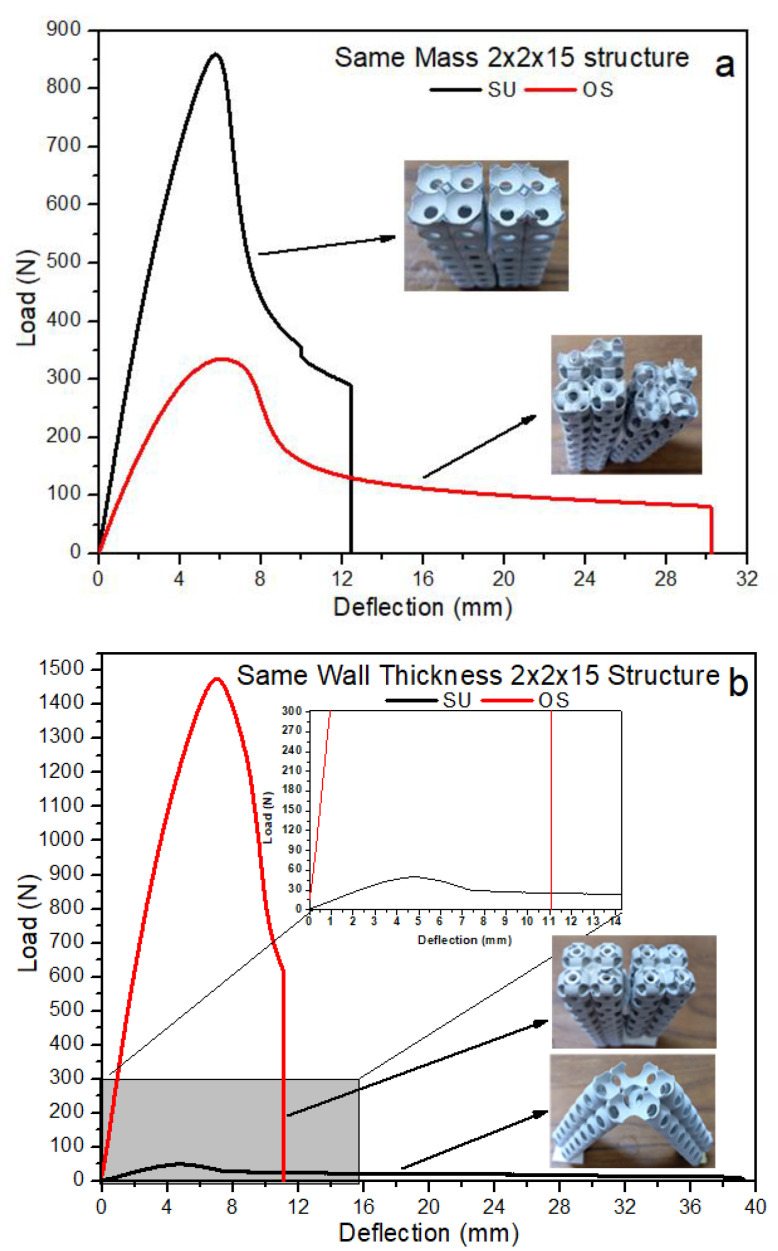
Critical buckling load of (**a**) same relative density column, (**b**) same wall thickness column.

The buckling of lattice structures depends on multi-parameters, such as wall thickness, tessellation, relative density, presence of column inside the structure, lattice pore size, and length of the column [37,46,47,48,49]. Figure 8 represents the local and global buckling behavior of all uniaxial compressive tested columns. For each type of sample, only one specimen is presented. The columns were tested compressively from top to bottom with the free moment in all directions to investigate the buckling directions. All four types of columns globally buckled from the center. According to Euler’s formula, the critical buckling load (Pcr) value is directly proportional to the second moment of inertia—placing the more material far from the neutral axis, the more Pcr will be exhibited by the column [50]. This study also proved that the more significant relative density column recorded the highest Pcr, while the lowest density column recorded the lowest Pcr, respectively. However, Pcr in these structures was investigated for strength-weight, failure mechanism, and post-buckling behavior. Figure 8 illustrates the mechanical behavior of SU and OS structures under the compression loading. As shown in Figure 8a, the SU-M structure showed a greater Pcr value; however, the post-buckling behavior is brittle. In contrast, the OS-M shows greater and flexible post-buckling behavior with a lower Pcr value. The OS-M column breaks apart at 30 mm, while it happens with SU-M at 12 mm.

Following the SU and OS 2 × 2 × 15 structure with M and T, Pcr behavior is represented in Figure 8b. A significant difference can be seen between the Pcr of SU and OS structures. It is because of the mass difference, as shown in Table 1. The OS-T is more flexible and exhibited a 15-folds higher force before starting to buckle. For example, OS-T initiated buckling at an 8 mm deflection, while 5 mm was recorded for the SU-T structure. Looking at comparison, the highest Pcr is recorded for OS-T—1450 N—followed by SU-M with around 850 N. Furthermore, the SU-T and OS-M also recorded a 320N and 85 N bearing force, respectively, before starting to buckle. Figure 8 revealed that having the same mass and bounding box, the SU-M is preferred to be used for greater Pcr. While considering the wall thickness, the OS is highly recommended for its Pcr value. Overall, lower mass structures showed better post-buckling with a lower Pcr value than the higher relative density columns. 

**Figure 9:**
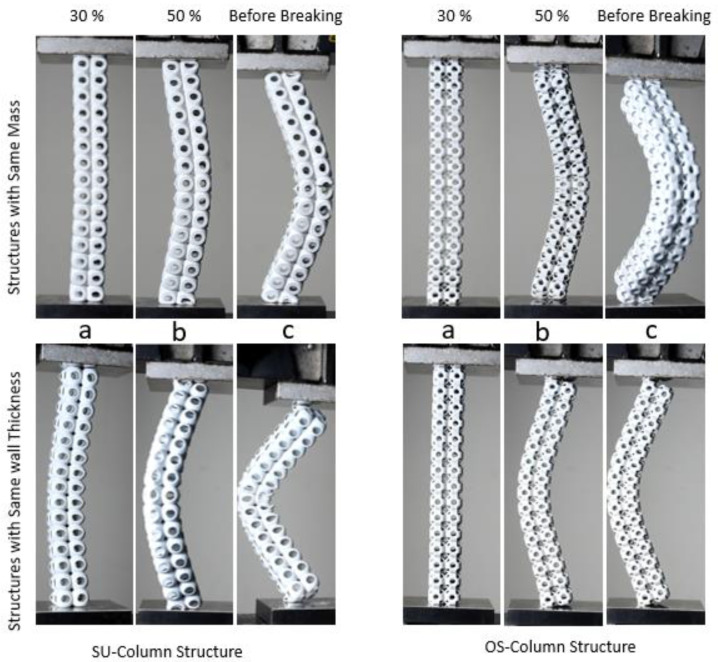

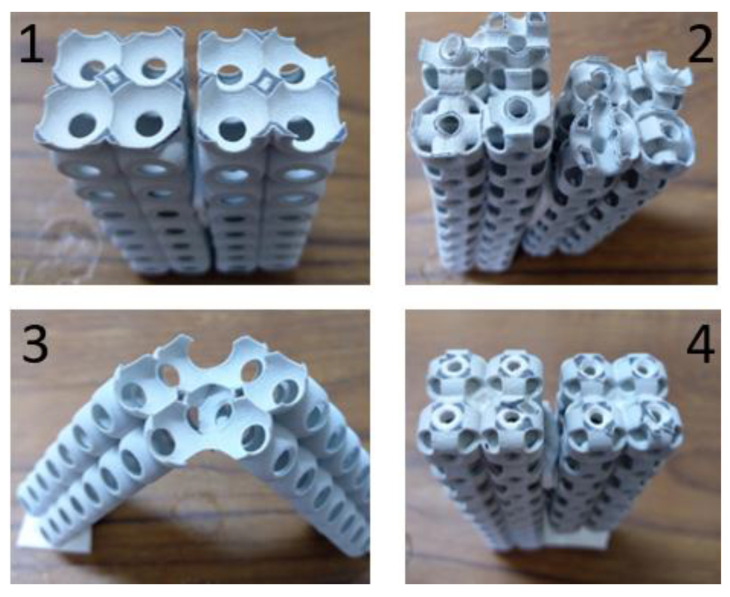
The figure (**above**) shows the overall buckling of each 2 × 2 × 15 structure under percentages of compression. While the (**below**) figures show an inner failure mechanism ((1) and (3) shows failure of SU structure while (2) and (4) shows the failure of OS structure) of the structure in which all (M and T) failed at the cell center, only OS-T is failed at the cell joints.

The failure mechanism of 2 × 2 × 15 structures is quite different when compared with 3 × 3 × 3 structures. Both SU samples of M and T are affected by local buckling. In Figure 9, a, b, the SU column’s local buckling shows that unit cells at the center of the column started squeezing with a progressively applied load before global buckling. Such cracks are propagated from the center of the SU-M and OS-M (see Figure 9). The OS-T failed from the unit cell’s joining section, as Figure 9 (4) shows. Therefore, keeping the same relative density and bounding box, SU-M structures are more likely to start local-buckling than OS structures. Additionally, the thickness of the wall is also highly influenced by post-buckling and cracks’ propagation. For example, having the lower wall thickness—OS-M and SU-T—enhanced the post-buckling by stretching the unit cell without tearing apart at the unit tessellation joint. It can be seen in Figure 9 that OS-M and SU-T structures are stretched significantly during the global buckling before falling apart. 

It was shown that all four types of 2 × 2 × 15 structures are globally buckled from the center. However, local buckling can only be seen in the SU structures due to topology characteristics. It is revealed that the global directional of buckling was affected in all the 2 × 2 × 15 structures by keeping relative density and wall thickness constant. In Figure 9, the structures with same mass buckled toward the right side, while same wall thickness structures buckled toward the left side. Based on these results, buckling direction can also be designed while keeping the relative density and wall thickness values. All the structures buckled from the middle with global buckling. Additionally, the post-buckling behavior also changed with the relative density of the structure. For example, the OS-T and SU-T hold the same wall thickness but differ in relative densities (see Table 1) Therefore, the SU-T with lower mass recorded the lowest Pcr value with significant post-buckling behavior.

### 3.3. Specific Strength

Lattice structures are recognized for their advantages of providing lightweight, shock resistant, stiff properties, and a specific strength. Specific strength is an essential mechanical factor for comparing structures with different masses or relative densities for their end applications. The specific strength is also called the strength-to-weight ratio of the material [51]. There are many ways to calculate it, but the most convenient is to divide the tensile or yield strength by its density or strength of materials by its structure mass. It is also documented that material with highly specific strength is more suitable for aircraft and automobile applications. Therefore, the specific strength of the 3 × 3 × 3 SU and OS structures and 2 × 2 × 15 structures were calculated at different relative densities and wall thicknesses. To calculate theoretically, the volume and density need to be calculated first, as mentioned in Equation (1) [51]. In Equation (1), the *V* represents the overall volume; *D*, the cylinder’s diameter; and *L*, denoting the cylinder’s length. However, in this study, the authors used the simple method of specific strength calculation, as mentioned in Equation (2) and [52]. This simple calculation method is meant to divide the strength of the material by its mass.

(1) $$V=\frac{\pi D^{2}}{4}L$$

(2) $$Specific\;Strength=\frac{Material\;\;\;strength}{Mass\;\;\;of\;the\;\;\;structure}\;$$

#### 3.3.1. Specific Strength of 3 × 3 × 3 Structure

**Figure 10:**
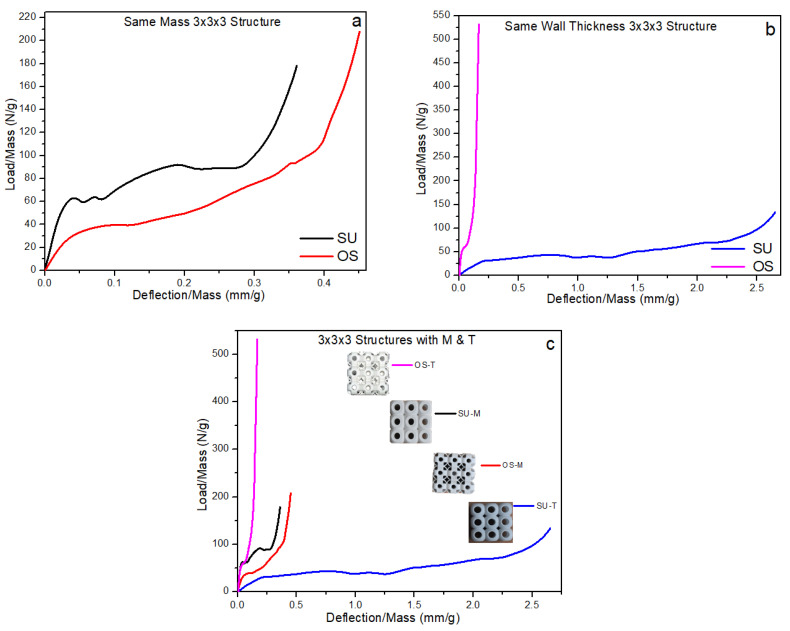
Specific strength of 3 × 3 × 3 lattice structures, (**a**) same density, (**b**) same wall thickness, (**c**) combined graph of both samples.

Figure 10 contains the specific strength graphs to investigate the influence of density and wall thickness on mechanical properties and revealed the specific strength by load dividing by mass. The bounding box of all the 3 × 3 × 3 structures remained the same, irrespective of the mass and wall thickness differences. This ratio indicates the mechanical properties’ relation with the mass and wall thickness of the structures. Despite the same density and same bounding box, the SU exhibits significant specific strength compared to the OS structure (see Figure 10a) because of four times lower wall thickness than the SU-M structure, as shown in Table 1. However, the OS-M has more potential to absorb compression energy.

The graph of the specific strength of the SU-M is fluctuated because of the random cell failure mechanism. For example, before the crack’s initiation, the ratio increased significantly up to 60 (N/g); however, the fluctuation started after cracking of the initial unit cell. This is followed by supporting the middle unit cell’s load to increase the ratio again. On the other hand, the OS-M recorded the overall lowest strength rate compared to the SU-M structure but maintained its physical shape with progressive compression. There are no cracks in the OS-M structure during compression; therefore, no fluctuations were observed in the graph. To summarize, these results reveal that having the same density and bounding box, the SU structure can be used for higher strength, while the OS can be recommended for high energy absorption. However, these results have some limitations, such as the SU, as compared to OS, has higher specific strength within a limited wall thickness. A separate study needs to be conducted to investigate these limitations and the mechanical properties’ relationship concerning wall thickness and density of each structure. These limitations and recommendations are lightly explained in Section 3.5.

The SU-T and OS-T’s specific strength trend is quite different from the SU-M and OS-M, as shown in Figure 10b. In graph b, the structures are investigated by keeping the wall thickness constant, irrespective of density. The OS-T showed very rigid behavior with the very highest specific strength compared to the SU-T structure. The OS-T structure’s specific strength is almost 16-folds—530 N/g at 0.3 mm/g—higher than the SU-T structure —120 N/g at 2.7 mm/g. This higher specific strength may be the reason for increasing the thickness of the wall. Alternatively, powder stuck in the OS-T structure could also be the reason. It can be concluded from these results that a slight increase in the wall thickness can significantly increase the specific strength of the OS structure. However, a lower wall thickness structure can also be recommended for higher energy absorption. The overall comparison of both simples with M and T and the same bounding box is represented by Figure 10c to investigate the specific strength. Overall, based on these results, the OS structure showed the highest specific strength compared to all other samples. It can also be seen that the rigidity of the OS structure is directly proportional to the wall thickness. At the same time, energy absorption can be achieved by keeping the structure’s relative density constant.

#### 3.3.2. Specific Strength of 2 × 2 × 15 Structure

**Figure 11:**
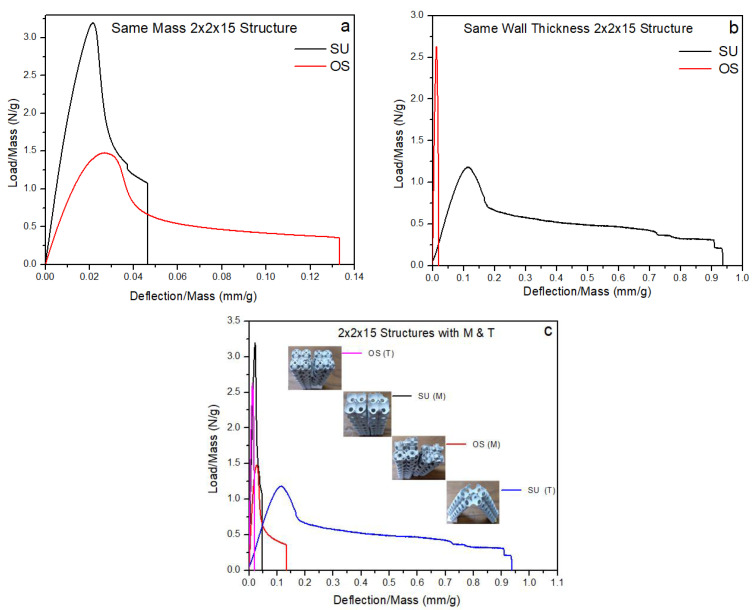
Specific strength of 2 × 2 × 15 lattice structures, (**a**) same density, (**b**) same wall thickness, (**c**) combined graph of both samples.

Compression of lattice structures leads to the linear buckling in the structure. This failure occurs when large transverse deformation is exhibited visibly in the structure and resistance to deformation rapidly decreases [53]. Usually, buckling failure occurs within elastic limit. However, a thin wall or wall thickness structure can lead the structure to fail before yield strength in the elastic region. Figure 11a represents the same mass; Figure 11b represents the same wall thickness and Figure 11c is describing the combined specific strength behavior of the 2 × 2 × 15 structures.

Figure 11a,b show the opposite behavior of the SU and OS structures. The SU structure recorded the highest specific strength with the same relative density, while the lowest value of specific strength is observed in the T structure. Similarly, the OS recorded the lowest specific strength value for the M structure, while the sharply highest value of specific strength can be seen for the T structure. In both cases, the lower specific strength value structure exhibited a comparatively higher energy absorption capacity. In contrast, the highest specific strength can be seen with very rigid behavior (see Figure 11c).

Based on Figure 11c results, the SU-M exhibited the highest specific strength value at 3.3 N/g and was followed by the OS-T with the second-highest value of specific strength at 2.7 N/g. Both structures are designed with greater wall thickness compared to their counterpart. Therefore, it can be concluded that the wall thickness of the structure can influence the specific strength of the 2 × 2 × 15 structure. In contrast, a 2 × 2 × 15 structure with lower wall thickness showed lower specific strength with comparative higher energy absorption for its counterpart. Hence, it can be concluded that comparing SU and OS structures, SU performance was better when the mass is critically considered. However, the OS structure may be more suitable for cases where lower energy absorption and high mechanical strength are essential. The difference in performance between SU and OS is significant and is evident in each graph.

### 3.4. Experimental Data Validation

**Figure 12:**
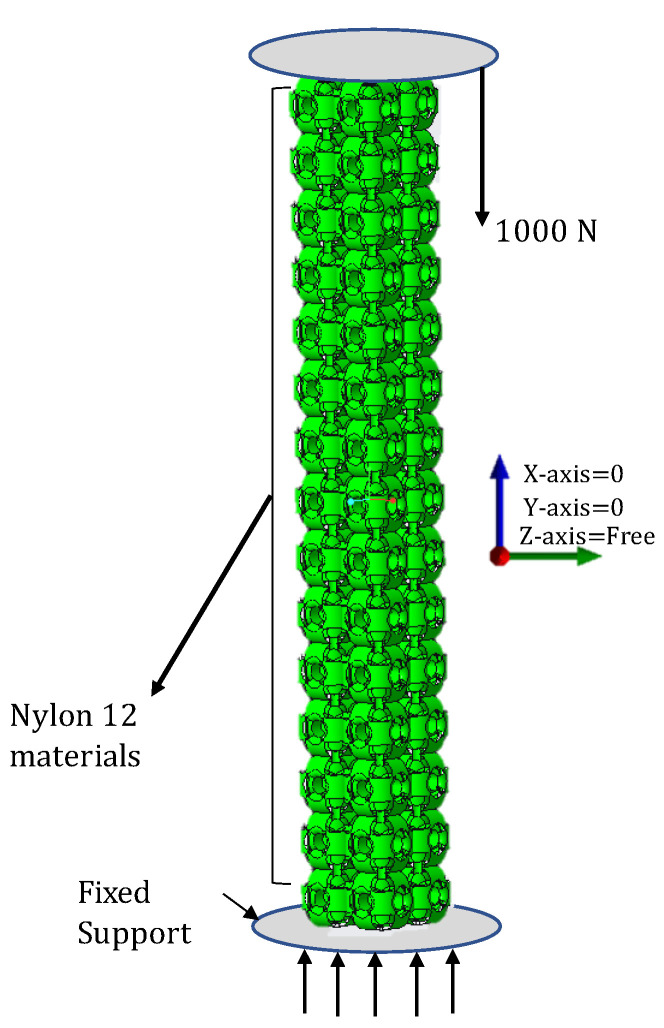
Numerical compression of the 2 × 2 × 15 structure with eigenvalue buckling analysis in ANSYS static structure.

Static structural and eigenvalue buckling analysis systems of the ANSYS 19.2 software (Ansys Inc., Canonsburg, PA, USA were used to predict the failure mechanism, strength, buckling, and critical buckling value of the structures. Boundary conditions were applied similarly as in experimental analysis, as shown in Figure 12. These structures were designed with nonlinear materials and geometric properties. Therefore, nonlinear data, as shown in Table 3, were added to the FEA materials section for both nonlinear and linear numerical analysis. All the structures were fixed at the bottom, whereas forces were applied on each structure’s top. Additionally, the structures were restrained at all directions and rotations except freely moving in the z-direction—downward. In return, structures were controlled from the x and y direction for movements. The 2 × 2 × 15 structure was subjected to deflection and load according to software limitations. The force option was only used as a critical buckling force. To restrain the slipping of the two surface areas, manual contacts were defined in between the two surfaces with the friction value of 2.0—as per PA 12 material standards—to transfer the load progressively.

**Figure 13:**
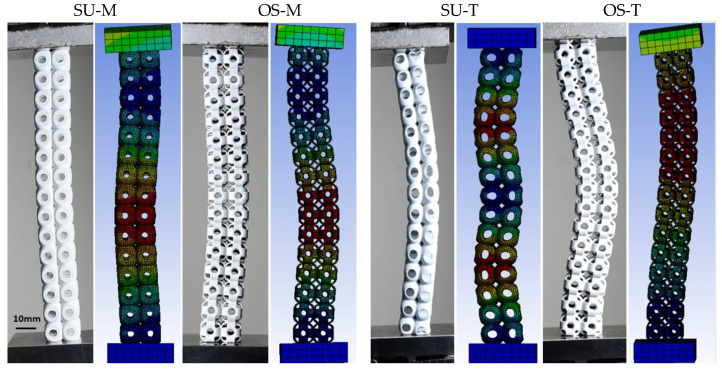
Comparison of experimental and FEA critical buckling load.

**Figure 14:**
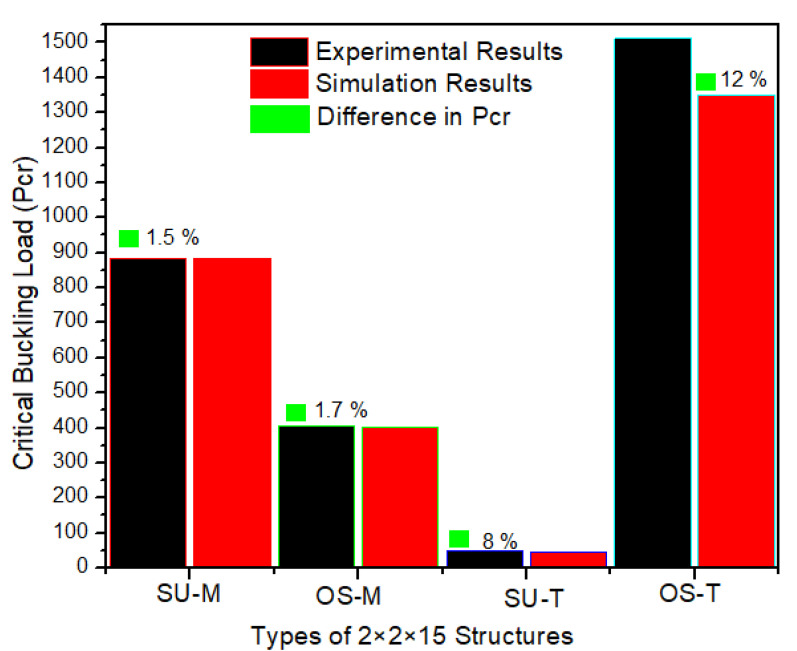
Experimental and finite element analysis of critical buckling load.

The numerically critical buckling load (Pcr) values are consistent with the experimental value, and no significant difference is recorded between them, as shown in Figure 13 and Figure 14. Only the column OS-T structure recorded a difference with a percentage of 12%. This difference occurred because the powders were stuck in the sample, as shown in Figure 7.

It can be seen from Figure 13 that both FEA and empirical results are following a similar trend. However, the difference in the results between the experiment and simulation can be found in Figure 14 with percentages. These differences may be the reason for boundary conditions, misalignment of loading, the variability of wall thickness, printing deficiency, and powder sticking issues [49,54,55].

It can be observed from Figure 13 that the physical buckling behavior of both empirical and FEA methods followed a similar pattern. The SU and OS with M and T are compared for critical buckling load, and fair agreements were found among experimental and simulation results. As a result, both similar results in the linear eigenvalue buckling analysis can be considered suitable for lattice structure. However, due to the finite element software limitation, the column’s post-buckling behavior was not considered.

It is revealed from the simulation and experimental results that lattice structure columns designed with different relative densities and wall thickness can influence the mechanical properties and buckling directions. To conclude, given these FEA and experimental results, the SU-M structure can be recommended to bear a higher buckling load where mass needs to be considered as minimum as possible. However, in the applications where a higher buckling load needs to withstand without consideration of mass: The OS-T can be recommended. Therefore, it is crucial to identify the optimum value of mass and wall thickness to the height of the structure and the objective of a specific dimension having the same materials, equal weight, and bounding box. The buckling failure can be minimized using this optimum value for each structure, considering their geometric structure for a specific application. Therefore, a completely experimental and analytical investigation is needed to specify these optimum values of each structure’s mass and wall thickness for a particular application. Some of the lattice structure columns’ parameters have already been studied and improved their critical buckling load [55,56].

### 3.5. Challenges and Recommendation

Additive manufacturing technology recently emerged, especially for the fabrication of complex structures. However, limitations are also observed during the additive manufacturing process. Among the primary challenges are anisotropy, high cost, mass production, warping, low accuracy, part size limitation, and powder sticking (see Figure 7) [57,58,59]. Powder trapping is a significant issue in most structures, especially when complex lattice structures are being fabricated [60]. However, this research area is not yet explored significantly by the researcher community. In return, this powder trapping limits the application of additive manufacturing for complex geometry, such as small-size close lattice structures, conformal cooling channels, and heat exchangers.

In this study, trapping of powder was also observed and affected the OS lattice structure’s mechanical properties. However, the experimental and analytical investigation of powder trapping in these lattice structures is beyond this study’s scope. Figure 7a,b show the trapped powder in the structures, while Figure 7c shows the powder’s quantity unstuck after the compression test. In this work, four types of the sample—SU-M, OS-M, SU-T, OS-T—were additively manufactured in powder bed fusion. However, the powder was only stuck in the OS-T structure due to the difference in wall thickness. Despite the same wall thickness of its counterpart—SU-T—no powders were trapped in the structure. Additionally, OS-M and SU-M structures are also free of powder, as shown in Figure 6. Therefore, it is verified that the OS has a limitation with additive manufacturing in terms of geometric structure. However, this limitation can be overcome with proper designing strategies.

**Figure 15:**
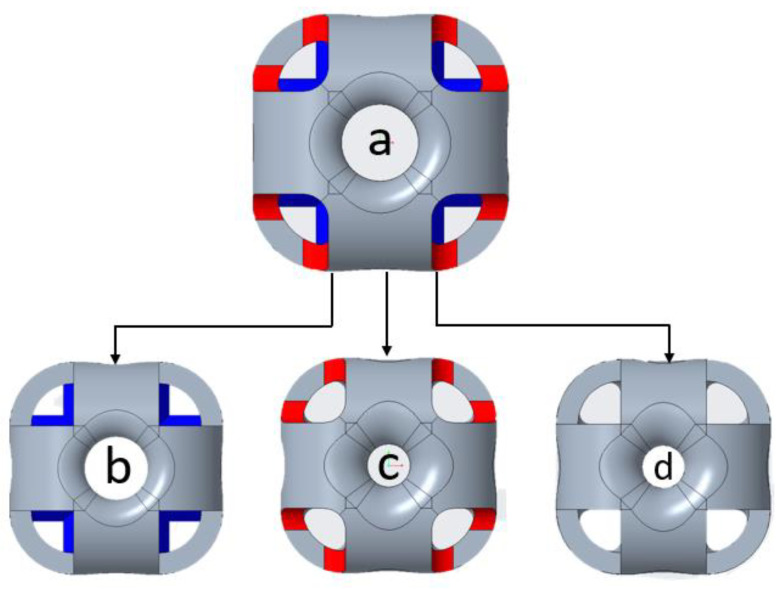
Recommended design strategies for OS-T structure to eliminate powder trapping.

The recommended OS-T structure with three steps from existing to a new one is illustrated in Figure 15. However, this is only a recommendation from the author; the fabricated and 3d structure may affect the mechanical properties, but determining these strategies’ effects is beyond this study’s scope. The main reason—as seen in the above figures—for the powder trapping was the wall thickness and rounds. Therefore, three steps were applied from (a) the existing structure, including (b) to remove or decrease the dimension of rounds for the structure as shown by the red color, (c) to reduce the diameter of the center holes (hollow column), and (d) to apply both: removing the rounds and decreasing the diameter of the central hollow columns. These changes in the structure can reduce the weight and mechanical properties; therefore, more mass can be added to the structure’s outer body. However, a complete investigation is needed to locate the mass, such as a concentrated and high-load area.

## 4. Conclusions

SU and OS lattice structures with the same relative density and wall thickness, having the same bounding box, were experimentally and numerically investigated. It was verified that the SU-M revealed better mechanical strength than the OS-M, with the value of force almost two-folds higher in the compressive response. In contrast, the OS-T recorded a 14-folds higher comparison strength to its counterpart (SU-T). Both cases demonstrate contrast energy absorption behavior: The OS-M and SU-T showed higher energy absorption than their counterparts. Compression behavior of OS structures was recorded as steady, and no cracks originated in the center of unit cells due to vertical columns. In contrast, cracks were produced in the SU structure during compression at the initial stages. It is revealed in both cases that SU structures were affected by local buckling that originated in the cracks from the center of unit cells. In contrast, the OS structure only failed under progressive compression. Moreover, the SU structure showed a greater Pcr value with brittle post-buckling behavior. Regarding Pcr, the OS-M revealed greater and more flexible post-buckling behavior with a lower Pcr value. The OS-T was more flexible and able to bear a 15-folds higher Pcr. Additionally, the post-buckling and crack propagations were highly influenced by the thickness of the structure wall. The OS-T showed very rigid behavior with the highest specific strength (16-folds) than the SU-T structure. It can be concluded from these results that a slight increase in the wall thickness can significantly increase the specific strength of the OS structure. Hence, the SU can be used in applications where being lightweight is a concern. In contrast, the OS column can be highly recommended in the application where only high strength needs to be considered regardless of mass consideration.

## Figures and Tables

**Table 1:** The complete dimension of all the samples.

Topology	Mass of a Unit Cell (g)	Wall Thickness (mm)	Dimensions of Structure(mm)			Relative Density of Designed Models (%)	Relative Density of AM Parts (%)
			Height	Width	Breadth		
SU-M (3 × 3 × 3)	0.27	2.3	30	30	30	28	29
OS-M (3 × 3 × 3)	0.27	0.65	30	30	30	28	25
SU-M (2 × 2 × 15)	0.27	2.3	149	20	20	28	27
OS-M (2 × 2 × 15)	0.27	0.65	149	20	20	28	24
SU-T (3 × 3 × 3)	0.127	1	30	30	30	13.5	10.5
OS-T (3 × 3 × 3)	0.39	1	30	30	30	39	45
SU-T (2 × 2 × 15)	0.127	1	149	20	20	13.5	10
OS-T (2 × 2 × 15)	0.39	1	149	20	20	39	40

**Table 2:** PA 12 material linear properties [42].

Density (g/cm^3^)	Young’s Modulus (MPa)	Poisson’s Ratio	Tensile Strength (MPa)	Ultimate Tensile Strength (MPa)
1.01	1437	0.33	27	44

**Table 3:** PA12 material non-linear properties [42].

**True stress** **(MPa)**	27.12	30.00	34.09	37.00	40.01	43.02	46.00	48.03	50.00	52.69
**True plastic strain (mm/mm)**	0	0.002	0.007	0.011	0.016	0.023	0.032	0.040	0.050	0.077

## Data Availability

Data sharing is not applicable to this article.
